# An outline of anemia among adolescent girls in Bangladesh: findings from a cross-sectional study

**DOI:** 10.1186/s12878-017-0084-x

**Published:** 2017-08-22

**Authors:** Sabuj Kanti Mistry, Fatema Tuz Jhohura, Fouzia Khanam, Fahmida Akter, Safayet Khan, Fakir Md Yunus, Md Belal Hossain, Kaosar Afsana, Md Raisul Haque, Mahfuzar Rahman

**Affiliations:** 1 Research and Evaluation Division, BRAC, BRAC Centre, 75 Mohakhali, Dhaka, 1212 Bangladesh; 2 Health, Nutrition and Population Programme, BRAC, BRAC Centre, 75 Mohakhali, Dhaka, 1212 Bangladesh; 3 College of Pharmacy and Nutrition, The University of Saskatchewan, 104 Clinic Place, Saskatoon, SK S7N 2Z4 Canada

**Keywords:** Adolescent anemia, Malnutrition, Hemoglobin, Socio-demographic factors

## Abstract

**Background:**

Anemia is a significant wide spread public health threat especially among the adolescent girls who are more vulnerable towards low level of hemoglobin particularly of low and middle income countries (LMICs). We investigated the prevalence of anemia among the adolescent girls (10–19 years) in Bangladesh and its socio-demographics distribution.

**Methods:**

We collected data digitally in ODK platform from a sub-sample of a nationwide cross-sectional survey of 1314 adolescent girls in 2015. Capillary blood hemoglobin level was estimated using HemoCue®; anthropometric measurements through standardized procedure and details socio-demographic information were captured and analyzed. Malnutrition was defined as BMI-for-age Z-score below -2SD (BAZ < −2SD), measured in WHO-AnthroPlus. Univariate analysis followed by multiple logistic regression were performed to examine the association between socio-demographic variables and anemia, while controlling the effect of potential confounding variables.

**Results:**

Overall, 51.6% girls were suffering from any form of anemia (non-pregnant-Hb < 12 g/dl; pregnant-Hb < 11 g/dl) while 46% were mildly (non-pregnant-Hb: 10–11.9 g/dl; pregnant-Hb: 10–10.9 g/dl) and 5.4% were moderately (Hb: 7–9.9 g/dl) anemic while only 0.2% were severely anemic. After controlling for relevant covariates in multiple logistic regression model, malnutrition (AOR: 1.42, 95% CI = 1.0–2.10, *p*-value = 0.083), non-pregnancy (AOR: 6.10, 95% CI = 2.70–13.78, *p*-value < 0.001), and households with bottom wealth quintile (AOR: 1.54, 95% CI = 1.03–2.30, *p*-value = 0.037) were identified as significant risk factors of anemia among adolescent girls of Bangladesh.

**Conclusions:**

Higher number of adolescent girls are still suffering from anemia in Bangladesh and non-pregnant adolescent girls contributed the most. Immediate, long term and sustainable public health intervention would require to combat the situation.

## Background

Anemia is a major public health problem affecting around 1.62 billion people globally [1, 2]. It is defined as a common blood disorders in which number of red blood cells, or the hemoglobin (Hb) concentration, falls below an established cut-off value, consequently impairing the capacity of the blood to transport oxygen around the body [3]. Anemia may develop at any stage of the life cycle [1] but children, adolescent girls and women of reproductive age are high risk groups for developing anemia [4, 5]. Anemia is a particular concern for adolescent girls i.e., aged 10–19 years [6], as this is a period of intense growth with higher iron requirement. This compounded with frequent menstrual blood losses and inadequate dietary iron intake in this period results in anemia [7]. Though anemia has multifaceted etiology, it primarily results from iron deficiency [3, 7, 8]. Worldwide, about 50% cases of anemia is caused by iron deficiency [9], but based on local conditions, this proportion may vary among population groups and areas [9, 10]. Some other haemopoietic micronutrient deficiencies like folate, riboflavin, Vitamins A and B_12_ may increase risk for anemia [7]. Infectious diseases such as malaria, tuberculosis and HIV/AIDS can also contribute to anemia, particularly prevalent in Africa and sub- Saharan Africa [4, 7]. Excessive blood loss resulting from hookworm infection and schistosomiasis can also lead to anemia [7, 11]. Some genetic or inherited hemoglobin disorders caused by inherited mutations of the globin genes leading to qualitative and quantitative abnormalities of globin synthesis (sickle-cell disease and thalassemia) also increases the risk for anemia, mainly in Mediterranean and Southeast Asian countries [4, 7, 12]. In Bangladesh more than 7000 children born each year with thalassemia and WHO report estimates that there are about 3% beta-thalassemia carrier and about 4% HB E/beta-thalassemia carrier in Bangladesh [13].

Anemia has serious consequences in adolescence with growth retardation [14] as well as impaired physical and cognitive performance [15]. Iron is also an essential nutrients for the functioning of neurotransmitter having a role in cognition, and in scarcity of hemoglobin, hypoxia develops with decreased cardiac output [16]. Several studies reported that iron supplementation among anemic adolescents and women had a role in cognition [17]. Higher behavioral disturbances along with reduced learning capacities and suboptimal school performance have also been documented among anemic school children [18]. Moreover, adolescent girls with anemia tend to commence their pregnancy with increased risk of morbidities and mortality for both mother and child [10].

Adolescent anemia is mostly prevalent in developing countries [4, 7, 19], and girls are more vulnerable than boys [20]. Studies identified that a large number of adolescent girls of South Asian region including Bangladesh are suffering from anemia along with other forms of malnutrition [21, 22]. Prevalence of anemia ranged from 31.6% to 99.9% among adolescent girls in India [2, 5] while in Nepal, the prevalence was as high as 68.8% [23]. Meanwhile, as of 2000, a study carried out in peri-urban areas of Bangladesh reported that the prevalence of anemia to be 27% among adolescent girls [24]. Recent study reported in 2016 that national prevalence of iron deficiency (serum ferritin level < 15.0 ng/ml) among non-pregnant non lactating women and children aged 12–14 years was 7.1% and 9.5% respectively in Bangladesh. It was found much higher (10%) among the children (12–14 years) in the rural Bangladesh. Furthermore, the study estimated that iron deficiency anemia could be much lower than anticipated among the Bangladeshi population of non-pregnant no lactating women (4.8%) and children aged 12–14 years (1.8%) [25]. However, there are still limited information till date in Bangladesh on the prevalence of anemia among adolescent girls (10–19 years) and its distribution. Other studies revealed the causal factors of anemia such consumption of traditional carbohydrate based diet lacking iron rich animal products, irregular eating habits [2], parent’s education [26], father’s occupation [27], age [19, 28], malaria [28], body mass index [19], menstruation ‘when marked as heavy’ [28], helminthes infestation [19, 28] and post-meal tea consumption [26]. However, all of these studies were carried out outside Bangladesh. This study will shed light on not only the number of adolescents suffering from low blood hemoglobin level but will also provide information on the factors associated with it.

## Methods

### Study site and participants

**Fig. 1:**
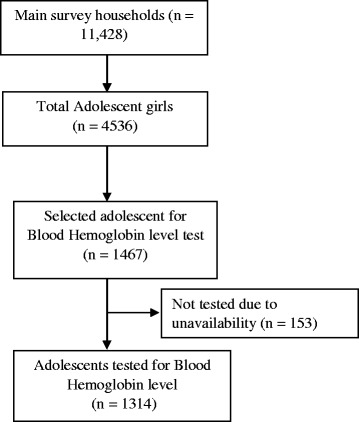
Sampling strategy and participants enrolment

The data used for this study was collected as a part of a large scale multistage cluster sampled nationally representative cross-sectional survey. The base survey was conducted among the 11,428 mothers of children aged less than 5 years between October 2015 to January 2016 by BRAC Research and Evaluation Division in collaboration with the BRAC’s Health, Nutrition and Population Programme. In the base survey, the sample was drawn from 21 equally divided clusters encompassing the entire country, of which 7 clusters were randomly selected for the present study. Thus, we opt to select all the adolescent girls (1467) from the selected households of 7 clusters as participants of the present study. However, we were unable to reach 10% of them due to unavailability and 1314 adolescent girls were finally selected. Inclusion criteria included was aged between 10 and 19 years, female and member of the surveyed households. The entire sampling strategy is summarized in Fig. 1.

### Data collection tools and techniques

Household level socio-economic and demographic information was collected through face-to-face interview of the targeted mothers, while other specific information pertaining to the adolescent girls were gathered by directly interviewing/measuring the girls. The entire data was collected electronically using ODK (Open Data Kit version 2.0), an android based open-source mobile platform software. ODK has easy interface that can be used in low-resource settings to support frontline health workers who has minimum education to collect data electronically. This easily customizable mobile app can work both online and offline, and has features like short message service (SMS) and GPS tracker which enable the real-time data collection monitoring. A total of 28 skilled female interviewers (having survey experience) were recruited for data collection. A fifteen-day intensive training were organized which included lectures, mock interviews, role play and field practice at the community level. A training manual was developed to guide the interviewers in the field. Several extensively trained teams of enumerators were formed for data collection each consisting of one supervisor and interviewers. The ODK was used in offline during the data collection and the questionnaires were uploaded every evening. The ODK server was hosted at the Head Office and was maintained by an experienced ICT person.

To ensure accuracy of information of such a large dataset, a number of quality control measures were undertaken at different stages of the data collection procedure. For example, a researcher statistician was position at the head office to check the live data and to provide feedback thereby. Considering the fact that our main respondents were women, we recruited experienced female field enumerators for data collection to minimize bias [29]. We established a multilayered monitoring system to validate, standardize and maintain data quality and performed tasks such as spot checking, thorough checking of the filled questionnaire in tablet, back checking and provided necessary feedback. Seven teams were working throughout the country headed by an experienced monitor for each, and researchers from headquarter also visited the survey areas frequently with the main task of supervision of data collection process.

### Variables assessed/measured

#### Wealth index

Construction of the wealth index was based on principal component analysis (PCA) of key socioeconomic variables. Each variable was given a weight based on its loading in the first general factor identified in the PCA. The resulting score for each household was then standardized with mean ‘0’ and standard deviation ‘1’ [30]. Households were then ranked and assigned a score ranging from 1 to 5 with 5 identifying the wealthiest households.

#### Hematological assessment

Capillary blood was collected from all participants through pricking the fingertips by lancet, while strict aseptic environment was maintained throughout the entire process. A separate lancet was used for each individual and hemoglobin (the oxygen carrying part of the blood cell) concentration was determined using the Hemocue portable hemoglobinometer (B-Hemoglobin Photometer, HemoCue AB, Angelhom, Sweden). A total of 7 machines were used and the enumerators were trained by a physician on the procedure and aseptic techniques. Researchers frequently visited the field and rechecked a number of adolescent girls for their Hb concentration to validate the enumerators’ activities. WHO cut-off point was used for classifying severity of anemia. Non pregnant adolescent girls with Hb level < 12 g/dl considered suffering from any form of anemia; 10.0–11.9 g/dl mildly anemic, 7.0–9.9 g/dl moderately anemic and <7.0 g/dl were considered severely anemic. For pregnant adolescent girls, the cut off value of any form of anemia was < 11.0 g/dl; mild anemia 10.0–10.9 g/dl, moderate anemia 7–9.9 g/dl and girls with Hb level < 7 g/dl were considered severely anemic [31].

#### Anthropometric measurement and calculation of BMI

Height was measured to the nearest 0.1 cm by using a board with an upright wooden base and a movable headpiece, on a flat surface. Weight was measured with an electronic scale at 0.1 kg precision. Body mass index (BMI), defined as weight in kg/(height in meter)^2^ is the preferred method to classify nutritional status of adults. However, since adolescence is a period of intense growth, both height and weight changes rapidly during this period. Thus, BMI-for-age Z-score (BAZ) is the recommended indicator for assessing nutritional status among adolescent girls aged 10–19 years [32]. The nutritional status of the selected adolescent girls was measured using BAZ through WHO Anthro Plus software. Malnourished (< −2SD), Well nourished (− 2SD to +1SD), and Over nourished (> +1SD) were estimated.

### Statistical analysis

Statistical analysis was conducted using STATA software (version 12.0). We used Chi-square test to assess the association between anemia and associated factors in contingency tables with a statistical significance level ≤ 0.05. Simple logistic regression (also called single covariate logistic regression) model was employed to analyze the potential risk factors for anemia among adolescent girls and crude odds ratio (COR) with 95% confidence interval (CI) were calculated. We followed the model building strategy as described by Akaike (1973) where the multiple logistic regression model with smallest Akaike Information Criteria (AIC) value considered as the best model [33].

## Results

### Background characteristics of the respondents

Table 1 presents detailed socio-demographics of the study participants. It was found that, nearly half of the respondents (47.9%) were aged between 10 and 14 years. Among them, around 18.9% and 17.5% of the adolescent girls belonged to the wealthiest and poorest households respectively. Majority (80.8%) of them were literate and mostly (85.5%) from rural areas. Nearly 60% of the girls were single and over one third (38.3%) were married before they reached 19 years, while 3.4% ware pregnant at the time of survey. Moreover, 20 of the 45 currently pregnant women were consuming Iron and Folic Acid (IFA) supplement tablets.

**Table 1:** Demographic and socioeconomic characteristics of adolescent girls

Characteristics	Frequency	Percentage (%)
	(Total *N* = 1314)	
Age category		
10–14 years	629	47.9
15–19 years	685	52.1
Wealth index		
Lowest	230	17.5
Second	265	20.2
Middle	276	21.0
Fourth	295	22.4
Highest	248	18.9
Literacy		
Literate	1061	80.8
Illiterate	253	19.2
Region		
Rural	1123	85.5
Urban	191	14.5
Marital status		
Single	811	61.7
Married	503	38.3
Currently pregnant		
Yes	45	3.4
No	1269	96.6

### Prevalence of anemia

Overall, 51.6% of the surveyed adolescent girls were found sufferings from any form of anemia. Most of them (46%) were suffering from mild form of anemia, followed by moderate anemia prevalence of 5.4%, and only a few of them (0.2%) were severely anemic. Mild anemia was comparatively higher among younger girls of age 10–14 years than older girls aged 15–19 years (48.3% versus 43.8%). However, moderate anemia prevalence was higher among older group (6.1%) than those of younger age (4.6%) (Data not shown).

### Socio-demographic distribution of anemia

Socio-demographic distribution of anemia is summarized in Table 2. Higher proportion (54.4%) of adolescent girls from lowest wealth quintile were suffering from anemia compare to those of the richest (47.2%). Not much difference was observed in anemia prevalence between single and married adolescent girls, roughly around 50% among both groups. Similar result was also noticed in terms of household food security and anemia status, prevalence varies around 51% among secured and unsecured households. Anemia prevalence was slightly higher among adolescent girls from urban slums compared to those from rural (53% versus 51%, *p*-vale = 0.589). Pregnancy status identified as a significant factor associated with anemia among adolescent girls. Nearly 16% of the pregnant adolescents were anemic while 47% of non-pregnant adolescents were anemic (*p*-value < 0.001). Also, among the malnourished adolescent girls (BMI-for-age Z-score < −2SD) 60% were anemic compared to around 50% of well-nourished girls (*p*-value = 0.051). Meanwhile, mean height and weight was 144.1 ± 16.1 cm and 39.5 ± 11.1 kg respectively and higher among non-anemic girls compared to anemic. It was also noted that mean hemoglobin level was 11.82 ± 1.15 g/dl, while it was 10.99 ± 0.9 g/dl and 12.7 ± 0. 61 g/dl respectively among anemic and non-anemic adolescent girls with a *p*-value <0.001 (Data not shown).

**Table 2:** Socio-demographic characteristics and anemia status among adolescent girls

Socio-demographic characteristics	Anemic	Total	Chi-square	*p*-value
	% (n)	n		
Total	51.6 (678)	1314		
Age				
10–14 years	53.3 (335)	629	1.333	0.248
15–19 years	50.1 (343)	685		
Household wealth quintile				
Lowest quintile (Poorest)	54.4 (125)	230	3.519	0.475
Second lowest quintile	54.3 (144)	265		
Middle quintile	50.7 (140)	276		
Second highest quintile	51.5 (152)	295		
Highest quintile (Richest)	47.2 (117)	248		
Region				
Rural	51.3 (576)	1123	0.292	0.589
Urban	53.4 (102)	191		
Marital status				
Currently single	52.7 (427)	811	0.94	0.332
Currently married	49.9 (251)	503		
Household food security				
Unsecured	51.8 (199)	384	0.0492	0.824
Secured	51.5 (479)	930		
Currently pregnant				
No	52.9 (671)	1269	24.237	<0.001^***^
Yes	15.6 (7)	45		
BMI for age				
Well nourished	50.8 (607)	1196	3.8137	0.05 1*
Malnourished	60.2 (71)	118		

^***^ *p* ≤ 0.01, ^*^ *p* ≤ 0.10

### Risk factors of anemia

In simple logistic regression analyses, malnutrition and pregnancy status were significantly associated with anemia among adolescent girls (Table 3). The crude odds ratio (COR) for malnutrition status with anemia is found as 1.5 with *p*-value 0.052 where the 95% confidence interval was found as (1.0, 2.2). Likewise, the COR for non-pregnancy compared to pregnancy status with anemia was found 0.2 with *p*-value < 0.001 where the 95% confidence interval was (0.1–0.4).

**Table 3:** Association of socio-demographic variables with anemia status

Characteristics	Crude			Adjusted		
	OR	*p*-value	95% CI	OR^a^	*p*-value	95% CI
Household wealth quintile						
Highest quintile (Richest)	1.0			1.0		
Second highest quintile	1.2	0.313	0.9–1.7	1.3	0.187	0.9–1.8
Middle quintile	1.2	0.417	0.8–1.6	1.3	0.154	0.9–1.9
Second lowest quintile	1.3	0.105	0.9–1.9	1.5	0.043**	1.0–2.2
Lowest quintile (Poorest)	1.3	0.118	0.9–1.9	1.5	0.041**	1.0–2.3
Region						
Rural	1.0			1.0		
Urban	1.1	0.589	0.8–1.5	1.3	0.119	0.9–1.9
Household food security						
Unsecured	1.0			1.0		
Secured	1.0	0.917	0.8–1.3	1.1	0.547	0.8–1.4
Marital status						
Single	1.0			1.0		
Married	1.1	0.332	0.7–1.1	0.9	0.650	0.7–1.3
Currently pregnant						
No	1.0			1.0		
Yes	0.2	<0.001***	0.1–0.4	0.2	<0.001***	0.1–0.4
BMI for age						
Well nourished	1.0			1.0		
Malnourished	1.5	0.052*	1.0–2.2	1.4	0.093*	1.0–2.1

^a^OR adjusted with girls age, ***p ≤ 0.01, **p ≤ 0.05, *p ≤ 0.10

However, after adjusting for potential covariates such as age, wealth index, marital status, regional effect and food security in multiple logistic model, malnutrition remained as an independent risk factors for anemia. Indeed, a malnourished adolescent girl had 40% more chance of anemia than a non-malnourished girl. It was also found that a pregnant adolescent girl had 5 times less chance of anemia compare to a pregnant girl. On the other hand, adolescent girls hailing from poorest or second poorest households were 50% more prone to become anemic compared to those from richest households. Besides, other covariates such as marital status, region and household food security status did not show any significant association with anemia status among adolescent girls.

## Discussion

Our study reports the prevalence of anemia and distribution of severity of anemia in different socio-demographic strata among adolescent girls of the age bracket of 10–19 years in Bangladesh. The study further examines the risk factors of anemia among them. While, the prevalence of all form of anemia were found 51.6%, most of them (46%) were mildly anemic and few (5.4%) were moderate with only 0.23% severely anemic. Our study also found that malnutrition, pregnancy status and wealth quintile are significant risk factors of anemia among adolescent girls in Bangladesh.

Prevalence of anemia reported in our study was similarly high to the most recent Bangladesh Demographic and Health Survey (BDHS) where the prevalence of any form anemia were 48.6%, while mild, moderate and severe anemia were 39.2%, 9.4% and 0.0% respectively among adolescent girls of 15–19 years [22]. Though the participants of that survey were married adolescent girls, the findings are comparable as very little difference was observed in terms of anemia status between married and unmarried adolescent girls in our study. Our study is unique in that sense as it covered nationally representative sample of adolescent girls regardless their all socio-demographic variables including marital status, and habitat. Several other similar studies [27, 34, 35] conducted in India also found that girls were mostly suffering from mild to moderate anemia.

Few other studies [24, 36–39] were carried out till date examining the prevalence and risk factors of anemia among adolescent girls in Bangladesh. Most of the studies were outdated and conducted in school setting, while a few were carried out at community level. A study [37] conducted among adolescent girls aged 11–16 years in rural community found that 43.0% of the girls were anemic, which indicates that the situation is as worse as it was previously. School based studies were carried out in urban [38] or per-urban [24] areas and anemia prevalence was ranged from 22 to 27%. Studies [16, 27, 40] conducted in community setting of India also found a very high prevalence of anemia among adolescent girls and it ranged between 48 and 85%. The prevalence was 30.9% in Afghanistan and 58.1% in Sri Lanka [41] and 23% in Iran [42]. Several other studies conducted in sub-Saharan Africa also found high prevalence of anemia among adolescent girls [43].

Several socioeconomic and physiological factors such as literacy [44], father’s occupation [27], lower socio-economic status [35], traditional and irregular feeding habit [2] and lower BMI [45] are reported as the most important factors associated with anemia among adolescent girls. Nutritional deprivation has long been identified as one of the most important causes of anemia, particularly among adolescent girls from underdeveloped countries [28]. Our study identified that malnutrition (BMI-for-age Z-score < −2SD) is a significant risk factor of anemia among adolescent girls. Studies conducted in similar setting also found that lower BMI is associated with higher rate of anemia among adolescent girls [23, 27]. However, contradictory to this, BMI had not significantly been associated with anemia among adolescent girls from rural India [40].

A number of studies affirmed the association between low socio-economic status and anemia among adolescent girls from low and middle income countries [35]. Studies conducted in Bangladesh also clearly demonstrated the influence of poor socio-economic status on higher anemia prevalence and reported that around half of the anemic girls belong to the low socioeconomic status group [36]. Present study pointed that girls from the poorest wealth quintile were more prone to become anemic compared to those from the richest wealth quintile. Adolescent girls of poor socioeconomic condition tends to consume less diversified diet [46] with lesser micronutrient content which might have resulted in lower BMI with concomitant higher prevalence of anemia.

In Bangladesh, adolescent pregnancy is a major social and health concern as 31% of adolescents aged 15–19 was childbearing mother [47]. We also found than non-pregnant adolescent girls were more prone to become anemic compared to that of the pregnant girls. This may mean that non-pregnant adolescent girls would require more attention from different stakeholders, however, prevalence of anemic among pregnant adolescent was still high.

Our study has several strengths over other studies of that kind. It followed a multistage cluster sampling procedure covering a wide region of both rural and urban areas and is generable to the entire population. The biochemical procedure of testing blood hemoglobin level to ascertain anemia is one of the most effective methods of measuring anemia at community setting compared to other methods such as clinical examination. Another advantage is as it is a community based study it provides a population level assessment of anemia prevalence especially severe anemia, which is unlikely to be detected through school-based studies where only school going adolescent are available [27].

However, the study was subjected to certain limitations. Although, the study identified prevalence of anemia it cannot be confirmed whether the anemia is resulting from iron deficiency, as we have no data on dietary iron intake or serum ferritin level. Also, the study was unable to confirm the types and causes of anemia without the data on RBC indices (MCV, MCH, MCHC) and blood plates, which can be measured using Hematology analyzers. Furthermore, as the data was cross-sectional in nature, temporal relationship between the socio-demographics and anemia should be interpreted with caution. However, it is estimated that at least half of anemia worldwide is due to nutritional iron deficiency anemia [10] and 32% anemia in Bangladesh is iron deficiency anemia [24]. However, other research reported a low prevalence of iron deficiency anemia among Bangladeshi women, most of them above the age of adolescence [8]. And although, Bangladesh has a nationwide program to distribute iron and folic acid for pregnant women, no such program exists for adolescent girls. We also did not assess energy, protein or other macro or micronutrient intakes. Studies identified that hookworm infestation is significantly associated with anemia in adolescent girls [19, 40]. But this study did not collect information regarding hookworm infestation. Further studies regarding these issues would enhance our understanding and therefore more work is needed to determine the cause of anemia in these adolescent girls to determine what effective interventions are warranted.

## Conclusions

Our study reaffirm the fact that anemia is a major problem for adolescent girls in Bangladesh which need to be given highest priority. Malnourished girls are highly vulnerable to anemia, most of which belong to the poor socioeconomic class. Currently there is actually no large scale intervention focusing on to control anemia among adolescent girls of the country. Thus, it is essential to initiate interventions to improve dietary iron intake along with measures to control infections and infestations which involves unusual blood loss resulting anemia.

## Abbreviations

AIDS: Acquired immune deficiency syndrome
AOR: Adjusted odds ratio
BMI: Body mass index
COR: Crude odds ratio
HIV: Human immunodeficiency virus
ODK: Open data kit
PCA: Principal component analysis
WHO: World Health Organization
